# Anticancer, antioxidant, and antibacterial activities of low molecular weight bioactive subfractions isolated from cultures of wood degrading fungus *Cerrena unicolor*

**DOI:** 10.1371/journal.pone.0197044

**Published:** 2018-06-06

**Authors:** Anna Matuszewska, Magdalena Jaszek, Dawid Stefaniuk, Tomasz Ciszewski, Łukasz Matuszewski

**Affiliations:** 1 Department of Biochemistry, Maria Curie-Skłodowska University, Lublin, Poland; 2 Clinical Oncology Ward, St John’s Cancer Center, Lublin, Poland; 3 Department of Paediatric Orthopaedics and Rehabilitation, Medical University, Lublin, Poland; University of Palermo, ITALY

## Abstract

The aim of this study is to investigate *in vitro* the anticancer, antioxidant, and antibacterial activities of three low molecular weight subfractions I, II and III isolated from secondary metabolites produced by the wood degrading fungus *Cerrena unicolor*. The present study demonstrated that the low molecular weight subfractions III exhibited the strongest inhibitory activity towards breast carcinoma cells MDA-MB-231, prostatic carcinoma cells PC3, and breast cancer cells MCF7 with the half-maximal inhibitory concentration (IC_50_) value of 52,25 μg/mL, 60,66 μg/mL, and 54,92 μg/mL, respectively. The highest percentage of inhibition was noted at a concentration of 300 μg/mL in all the examined tumor lines. A significant percentage (59.08%) of ex-LMSIII inhibition of the MDA-MB-231 tumor line was reached at a concentration of 15 μg/ml, while the concentration applied did not affect normal human fibroblast cells. The low molecular weight subfraction III was the most effective and additionally showed the highest free radical 1,1-diphenyl-2-picryl-hydrazyl scavenging activity (IC_50_ 20.39 μg/mL) followed by the low molecular weight subfraction I (IC_50_ 64.14 μg/mL) and II (IC_50_ 49.22 μg/mL). The antibacterial activity of the tested preparations was evaluated against three microorganisms: *Bacillus subtilis*, *Staphylococcus aureus*, and *Escherichia coli*. The MIC minimal inhibitory concentration (MIC) values for the low molecular weight subfraction I, II, and III showed a stronger inhibition effect on *S*. *aureus* than on *B*. *subtilis* and *E*. *coli cells*. The MIC values for the low molecular weight subfraction II against *S*. *aureus*, *B*. *subtilis*, and *E*. *coli* were 6.25, 12.5, and 100 mg/mL, respectively.

## Introduction

Medicinal mushrooms have long been used in Asian countries due to their bioactivities such as anticancer, antioxidant, antimicrobial, hepatoprotective, antineurodegenerative, antidiabetic, antiangiogenic, and hypoglycemic effects in animals and in humans [1–4]. Of all known species of mushrooms, 650 have documented medicinal properties but, surprisingly, only approximately 20 are in common clinical use at present [5]. Some edible mushrooms, e.g. *Grifola fron dosa*, *Lentinus edodes*, *Flammulina velutipes*, *Pleurotus ostreatus*, *Tremella mesenterica*, and *Hericium erinaceus*, are used for medicinal applications. There are also some non-edible mushroom species used solely for medicinal purposes, for instance *Ganoderma lucidum*, *Schizophyllum commune*, or *Trametes versicolor*. Bioactive substances from fungi can generally be divided into two groups of high molecular weight compounds, which include primarily polysaccharides and proteins and low molecular weight compounds, such as indoles, terpenoids, or phenols. The second group comprises low-molecular-weight secondary compounds that can penetrate the cell membrane and act as effectors of specific signal transduction pathways [4–6]. Substances belonging to both groups have great medical potential. In addition to their nutritional values, they possess antitumor, antibacterial, antiviral, and immunomodulatory activities [7–9]. One of the most widely investigated groups of preparations isolated from higher fungi (Basidiomycota), especially wood degrading species, are enzymes involved in degradation of the lignocellulose complex, where oxidative and hydrolytic enzymes cooperate, including laccases, peroxidases and other oxidases, (hemi)cellulases, and different glycosidases [10,11]. Bioactive proteins constitute another important type of functional components in mushrooms with an increasing potential pharmaceutical value [12]. Hu et al. (2011) demonstrated that the fruiting bodies of the mushroom *Agrocybe cylindracea* produce a laccase with HIV-1 reverse transcriptase inhibitory activity and antiproliferative activity against HepG2 cells and MCF7 cells [13]. Another very well-studied group of fungal bioactive compounds comprises polysaccharides [14–16]. Natural extracts obtained from mushrooms have been used for many years for different health purposes. Aqueous extracts of *Funalia trogii* have been shown to have *in vitro* and *in vivo* anti-tumor efficacy [17]. Other authors reported cytotoxic and mutagenic effects of *F*. *trogii* and *C*. *versicolor* extracts on the HeLa cervical cancer cell line and human fibroblast cells [18]. It has also been reported that ethanol extracts from *Ramaria flava* exhibit a wide range of anticancer, antioxidant, and antibiotic activities [4]. Many substances isolated from fungi have been described as supplements to full mushroom extracts. Liu et al. (2006) isolated a xylose-specific lectin with antimitogenic and antitumor activities from fresh fruiting bodies of *Xylaria hypoxylon* [19]. It has been reported that lectins isolated from *Pholiota adiposa* and *Ganoderma microsporum* exhibited antiviral and antitumor activities as well [20–21]. Mushrooms are also a very efficient source of many bioactive phenolic substances, e.g. phenolic acids, flavonoids, hydroxybenzoic acids, hydroxycinnamic acids, lignans, tannins, stilbenes, oxidized polyphenols, and terpenoids [22–23]. It has been found that many phenolic compounds are very effective free-radical scavengers or metal inactivators [24]. Antioxidant compounds, i.e. phenolics, polysaccharides, tocopherols, flavonoids, carotenoids, glycosides, ergothioneine, and ascorbic acid, are found in fruiting bodies, mycelium, and cultures fluid [25]. Interestingly, the antioxidant potential in mushrooms is higher than in the most commonly used vegetables and fruits. Free radicals are known to induce oxidative damage in physiologically important biomolecules and play an important role in processes of aging, progress of cardiovascular diseases, cancer, impaired immune system, or inflammatory diseases [26]. Some authors have described isolation of various extracellular polyphenols with antioxidant properties, e.g. from the culture broth of *Inonotus xeranticus*, *Phellinus linteus* [27], and *Ramaria flava* [4].

For many years, *C*. *unicolor* was regarded as a nonedible fungus and was intensively studied as an efficient producer of extracellular laccase produced in noninduced conditions of growth [28–29]. The results presented in our earlier paper show that laccase and total ex-LMS (extracellular low molecular weight secondary metabolites) produced by *C*. *unicolor* species possess cytotoxic and antiproliferative activity against cervical cancer cells (SiHa and CaSki) and melanomic cells [30–31]. The cytotoxic activity of laccase preparations towards several hematological malignancies has been described as well [32]. *C*. *unicolor* cultures are a very promising source of other bioactive substances, not only laccase, whose properties have been partially determined [33].

The aim of the present report will be to evaluate the antitumor, antioxidant, and antibacterial activities of three subfractions separated from the total ex-LMS produced by *C*. *unicolor*.

## Materials and methods

### Mushroom growing conditions and separation of fungal samples

*Cerrena unicolor* (Bull. ex Fr.) Murr. was obtained from the culture collection of the Regensburg University and deposited in the fungal collection of the Department of Biochemistry (Maria Curie-Sklodowska University, Poland) under the strain number 139 (ITS sequence deposited in GenBank under accession number DQ056858) [34]. The fermentor scale cultivation was performed at 26°C in a 2.5 L Bioflo III (New Brunswick Scientific, New Brunswick, NJ, USA) fermentor containing 2 L of a sterilized Lindenberg and Holm medium optimized as described by Janusz et al. in [29]. The medium in the fermentor was inoculated with crumbled fungal mats (10% of the total medium volume), aerated, and stirred at 100 rpm. The onset of the idiophase (the stage of the production of secondary metabolites) was determined as recommended by Jennings and Lysek [35]. 10-day-old idiophasic cultures were harvested and filtered through Miracloth (Calbiochem). The biomass and culture fluid obtained were used for further assays. The culture liquid obtained after mycelium separation was centrifuged at 10.000 ×g for 15 min. The supernatant was immediately subdivided into two fractions on the ultrafiltration system Pellicon 2 Mini holder (Millipore, Bedford, MA, USA) with an Ultracel mini cartridge (10 kD cut-off). The starting fraction containing compounds with a molecular weight below 10 kDa had already been used as a source of low molecular weight metabolites (extracellular low molecular weight subfraction, ex-LMS) and had been tested as described in our previous publication [32]. The antioxidant and pro-oxidative properties, the antibacterial, antiviral, immunomodulatory, and anticancer activities, and the toxicity of the ex-LMS fraction have also been presented in previously published reports [30–33]. In this study, a fraction containing compounds below 10 kDa was fractionated on a Sephadex G-10 column (20 cm x 2 cm) into two subfractions (below and above 700 Da)–ex-LMSI and ex-LMSII. Additionally, the same fraction (below 10 kDa) was precipitated using an ammonium sulfate-saturated solution for isolation of low molecular weight proteins and dialyzed against 4 L of distilled water at 4°C–ex-LMSIII. The content of ammonium sulfate was checked with the BaCl test. The three ex-LMSI, ex-LMSII, and ex-LMSIII fractions were subsequently lyophilized and used as a source of natural low molecular weight metabolites.

### Biochemical analysis

#### Determination of proteins, carbohydrates, and phenolic compounds

Protein concentrations were determined using the Bradford reagent and bovine serum albumin as a standard [36]. The total content of the phenolic compounds was determined with diazosulfanilamide using the DASA test [37], where the absorbance was measured at 500 nm and vanillic acid was used as a standard. The total carbohydrate content was determined by the phenol-sulfuric acid assay with D-glucose as a standard [38].

#### FT-IR spectroscopy analysis of ex-LMS samples

The analyses of ex-LMSI, ex-LMSII, and ex-LMSIII were performed using lyophilizates. FTIR spectroscopy was performed with a spectrometer (Thermo Scientific Nicolet 8700A with FT Ramana Nicolet NXR module) in the wavelength range 4000–400 cm^−1^.

### Antioxidant properties

#### Free radical 1,1-diphenyl-2-picryl-hydrazyl (DPPH)-scavenging test

The total antioxidant capacity of the three fractions was determined using the DPPH radical as a reagent, according to the procedure described by Paduch et al. [39]. This method is based on the ability of 1,1-diphenyl-2-picrylhydrazyl (DPPH) to decolorize in the presence of antioxidants. Subsequently, 100 μL of the test compound at concentrations ranging from 6.25 to 800 μg/mL were mixed with 0.1 mL of the DPPH solution (0.2 mg/mL in ethanol) and the absorbance at 515 nm was determined after 2, 5, 10, 15, 20, and 30 min of incubation at room temperature. Trolox and ascorbic acid (Vit. C), i.e. the well-known standards with strong antioxidant activities, were used as positive controls. The percentage of inhibition of DPPH oxidation was calculated according to the following formula:

$$DPPH\_scavenging\_effect(\%)=\frac{A_{control}-A_{sample}}{A_{control}}\times 100$$

where A_c_ means the absorbance of the control sample andA_t_ means the absorbance of the standard or tested compound. The antioxidant ability of the sample was expressed as IC_50_.

#### [2,2’-azinobis-(3-ethylbenzothiazoline-6-sulfonic acid)] (ABTS) radical-scavenging test

The ABTS radical-scavenging activities of the fractions were determined using the method of van den Berg et al. [40], Duo-Chuan [41], and Re et al. [42] with modification. A stock solution was prepared by dissolving 7.4 mM ABTS and 2.6 mM potassium persulfate in MQ water. After 16 h, the concentrated ABTS stock solution was diluted with phosphate buffered saline (PBS) pH 7.4 to absorbance recorded at 734 nm. Subsequently, 10 μL of the analyzed compound at concentrations ranging from 6.25 to 800 μg/mL were mixed with 990 μL of the ABTS radical solution and the absorbance was measured. The percentage of inhibition of ABTS oxidation was calculated using the following formula:

$$ABTS^{•+}\_scavenging\_effect(\%)=\frac{A_{control}-A_{sample}}{A_{control}}\times 100$$

where A_control_ means the absorbance of the control and A_sample_ is the absorbance at 734 nm of the tested compound/standard. A Trolox and ascorbic acid calibration curve was prepared for a concentration range from 6.25 to 800 μg/mL and IC_50_ values were obtained.

#### Hydroxyl radical-scavenging activity assay

The OH radical-scavenging activity assay was conducted according to the Fenton method [43–45] with some modifications. 100 μL of the sample were incubated with a mixture containing 20 μL of FeSO_4_ · 7H_2_O (9 mM), 20 μL of a hydroxybenzoic acid solution with ethanol (9 mM) and 20 μL of H_2_O_2_ (8.8 mM) in a 37°C water bath for 30 minutes. The percent OH radical-scavenging effect of each sample was calculated using the following equation:

$$OH\_scavenging\_activity(\%)=\frac{A_{control}-A_{sample}}{A_{control}}\times 100$$

where A_control_ is the absorbance of the control reaction and the sample is replaced by 100 mL ethanol. The tests were performed in triplicate.

### Analysis of the antibacterial activity

#### Inhibitory zone assay

*Staphylococcus aureus* (ATCC 6538), *Escherichia coli* (ATCC 25922), and *Bacillus subtilis* (ATCC 6633) bacterial strains were used as indicator bacteria and inoculated into a commercially available Muller-Hinton Agar II medium (LabM (TM), IDG plc, UK), 38g/L, with the inoculum solution (100 μL) of ca. 1×10^5^ CFU/mL of each kind of microorganisms smeared on the standard assay medium. 100 μL of the specimen (concentration 1 mg/mL) were added into the agar well in the center of Petri dishes and left to incubate for 2 hours at room temperature; afterwards, the plates were transferred to 37°C for 18 h. Sterilized physiological saline was used as a control. After the incubation of inoculated bacterial cultures treated with ex-LMSI, ex-LMSII, and ex-LMSIII, the inhibition zones were measured.

#### Minimal inhibitory concentration (MIC)

The minimal inhibitory concentration (MIC) of ex-LMSI, ex-LMSII, and ex-LMSIII was evaluated using the two-fold serial dilution method [46]. The samples were dissolved in methanol (filtered, 0.22 μm) and then diluted to obtain 200 mg/mL stock solutions. 0.5 mL of the stock solution was incorporated into 0.5 mL of sterilized nutrient broth for bacteria and serially diluted to achieve 100, 50, 25, 12.5, and 6.25 mg/mL, respectively. A 100-μL aliquot of the standardized suspension of the test bacteria (10^5^CFU/mL) was transferred to the wells of a 96-well tissue culture plate. Then, another 100 μL of diluted samples were added to each well and the inoculated 96-well tissue culture plates were incubated at 37°C for 24 h. The MIC was defined as the lowest concentration of samples inhibiting the visible growth of the tested microorganisms.

### Anticancer assay

The test sample was subjected to the MTT assay to determine the *in vitro* cell growth inhibitory activity against the human cancer cell lines (MCF7, MDA-MB-231, and PC3) [47]. The cells were grown in tissue culture flasks in RPMI 1640 medium at 37°C in an atmosphere of 5% CO_2_ and 100% relative humidity in a CO_2_ incubator. A 100-μL aliquot of cells (10^5^ cells/mL) was transferred to the wells of a 96-well tissue culture plate. The cells were allowed to grow for 12 h and then they were treated with the sample. 100 μL of the test samples (300, 150, 15, 1.5, and 0.15 μg/mL) were added to the wells, and the cells were further incubated for another 48 h at 37°C in an atmosphere of 5% CO_2_. 20 μL of MTT (5 mg/mL in phosphate-buffered saline) were then added to each well and the cells were further cultured for 4 h. After removal of the medium, 100 μL of dimethyl sulfoxide (DMSO) was added to each well [4]. The absorbance was measured on a microplate reader (Thermo LabSystems, Grand Rapids, OH, USA) at the wavelength of 570 nm [26]. Suitable blanks and a positive control were also included, and paclitaxel was used as the positive control. The inhibition percentage was calculated using the following formula:

$$\begin{array}{l} Inhibition\_activity(\%)=\frac{A_{control}-A_{sample}}{A_{control}}\times 100 \end{array}$$

where A_control_ is the absorbance of the control reaction and A_sample_ is the absorbance in the presence of the sample. Each test was done in triplicate and the concentration required for 50% inhibition of viability (IC_50_) was determined.

### Statistical analysis

All results presented in the paper are expressed as mean and standard deviation (±SD) from three experiments (n = 3). Differences in mean values between the groups were analyzed by a one-way analysis of variance (ANOVA) with post-hoc Tukey HSD test and all tests were considered statistically significant at p ≤ 0.05.

## Results and discussion

*C*. *unicolor* represents wood degrading fungi from the phylum Basidiomycota, which are exceptionally useful biotechnological tools in various industrial processes. In recent years, *C*. *unicolor* has been extensively studied as a very efficient source of extracellular laccase, which is an enzyme used on a wide scale in various industries. Recently, our results have indicated that laccase (LAC), crude endopolysaccharides (c-EPL), and the extracellular low molecular weight subfraction (ex-LMS) exhibit pro- and antioxidant properties as well as immunomodulatory, antibacterial, antiviral, and anticancer activities [30–33]. In this study, we tested the antioxidant, antibacterial, and cytotoxic activities of three subfractions of low molecular weight metabolites: ex-LMSI, ex-LMSII, and ex-LMSIII derived from the total *C*. *unicolor* ex-LMS preparation.

### Characterization of the biochemical properties of ex-LMSI, ex-LMSII, and ex-LMSIII

*C*. *unicolor* is a source of natural active low molecular weight metabolites. Our analysis focused on three fractions of extracellular low molecular weight secondary metabolites from *C*. *unicolor* cultures (ex-LMSI, ex-LMSII, and ex-LMSIII). Our earlier study of the chemical composition of ex-LMS from *C*. *unicolor* showed the presence of sugars (total carbohydrates: 780.07 μg/mL, reducing sugars: 507.14 μg/mL, total polysaccharides: 272.93 μg/mL), proteins (189 μg/mL), and phenolic compounds (15 μM) [33]. The analysis of the chemical composition (concentrations of proteins, total carbohydrates, and total phenolic compounds) of idiophasic ex-LMSI, ex-LMSII, and ex-LMSIII isolated from *C*. *unicolor* revealed distinct differences between the investigated preparations (Table 1). The ex-LMSIII fraction contained an evidently higher amount of proteins (43.7 μg/mL) than ex-LMSI (2.25 μg/mL) and ex-LMSII (4.87 μg/mL). The concentration of phenolic compounds was significantly higher in ex-LMSI (20.56 μM) than in ex-LMSII (4.45 μM) and ex-LMSIII (5.8 μM). The concentration of total carbohydrates was the highest in ex-LMSI, i.e. 36.82 μg/mL. Other authors showed the presence of the analyzed groups of compounds in full mushroom extracts obtained using organic solvents [48]. The presence of phenolic compounds, such as quercetin, chrysin, and pinocembrin, in water extracts from *Ramaria flava* has been confirmed in other investigations [4].

**Table 1:** Chemical composition of ex-LMSI, ex-LMSII, and ex-LMSIII isolated from *C*. *unicolor*. Yield of total carbohydrates, concentration of phenolic compounds, and protein content. The samples of ex-LMS were dissolved in distilled water (1 mg/mL) and used for the tests.

Samples	Protein (μg/mL*)	Total carbohydrate (μg/mL*)	Total phenolic compounds (μM)
ex-LMS I	12.25 ± 4.3a	36.82 ± 4.2a	20.56 ± 1.6a
ex-LMS II	4.87 ± 0.6b	29.35 ± 1.4b	4.45 ± 0.4b
ex-LMS III	43.7 ± 2.6c	5.48 ± 0.7c	5.8 ± 0.4b

All results are expressed as mean ± SD from three experiments (n = 3). Values with different letters within the columns are significantly different (p ≤ 0.05).

* μg of substances for 1g of dry mass of lyophilized preparation.

**Fig 1:**
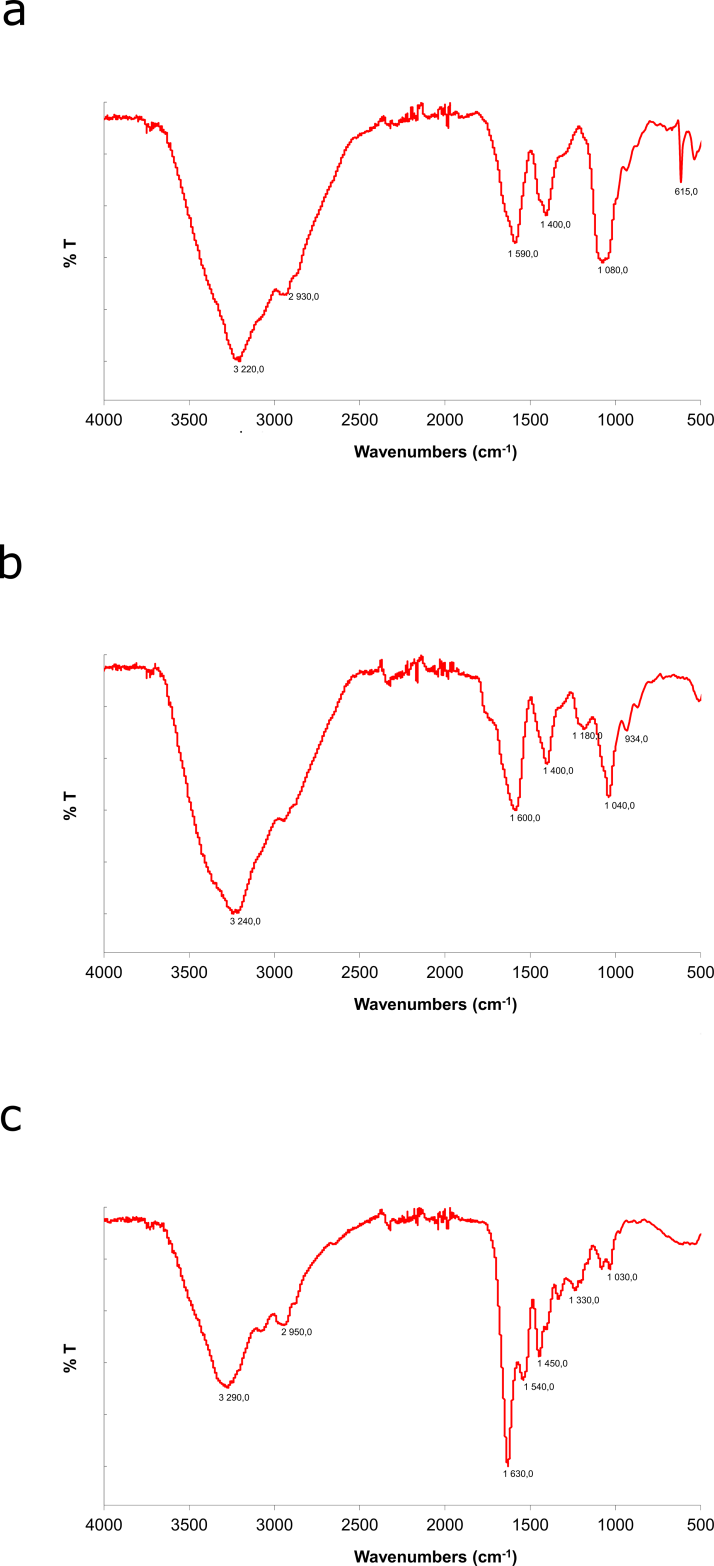
FT-IR analysis (FT-IR spectra of ex-LMSI (a), ex-LMSII (b), and ex-LMSIII (c) isolated from *C*. *unicolor*). FTIR spectroscopy was performed with a spectrometer (Thermo Scientific Nicolet 8700A with FT Raman Nicolet NXR module) in the wavelength range 4000–400 cm^−1^.

The FT-IR spectrum analysis of the ex-LMS subfractions of the *C*. *unicolor* idiophasic cultures demonstrated an aminoglycoside substance pattern (Fig 1). The characteristic strong broad band ca. 3200 cm^−1^ indicates the presence of OH stretching in hydrogen bonds [14]. The absorption bands between 1600 and 1400 cm^−1^ are attributed to the stretching vibration of the C–O bond of the carboxyl group, characteristic for proteins [49]. The band ca. 1000 cm^−1^ indicates the presence of O substituted glucose residues and β-linkages in the glucosidic chain [49]. The absorption band at 500–600 cm^−1^ suggests that the ex-LMS fraction contains pyranose rings in its structure. Analysis of the FT-IR spectrum of the ex-LMSIII fraction (c) showed higher absorption for proteins at ca. 1600 cm^-1^ and lower at ca. 3200 cm^-1^ compared to the ex-LMSI (a) and ex-LMSII (b) fractions. Similarly, the absorption at 1000 cm^-1^ is lower in the case of the ex-LMSIII fraction than for ex-LMSI and ex-LMSII.

### Antioxidant properties of ex-LMSI, ex-LMSII, and ex-LMSIII

Since our previous analyses showed that the scavenging ability of ex-LMS was at the same level or even higher in comparison to the model antioxidant substances, i.e. Trolox and ascorbic acid [33], we also checked the antioxidant capacity of the subfractions prepared in this study. In this research, we determined the scavenging properties of ex-LMSI, ex-LMSII, and ex-LMSIII using three methods: DPPH, ABTS, and OH radical scavenging assays. The analysis revealed strong reducing activity of these subfractions. The scavenging activity of the fractions tested was compared to those of ascorbic acid and Trolox. The IC_50_ values were 63.69 μg/mL for Trolox and 41.25 μg/mL for ascorbic acid in the DPPH method, 42.11 μg/mL and 28.23 μg/mL in the the ABTS method, and 54.61 μg/mL and 39.25 μg/mL in the the OH method, respectively. The DPPH radical-scavenging capacity of the analyzed samples is shown in Table 2. The activity can be evaluated by determination of the IC_50_ values, which correspond to the concentration of fungal samples that are able to scavenge 50% of free radicals present in the reaction mixture. High-IC_50_ values indicate low antioxidant activity. Ex-LMSIII shows the highest DPPH radical-scavenging activity, followed by ex-LMSI and ex-LMSII. The IC_50_ values of the samples were 20.39, 64.14, and 49.22 μg/mL, respectively. The measurements of the ABTS test showed slightly lower scavenging capacity of the ex-LMS fractions (in particular ex-LMSIII) in relation to the DPPH method (Table 2). The IC_50_ values in the ABTS scavenging method were 81.12 μg/mL (ex-LMSI), 39.78 μg/mL (ex-LMSII), and 31.49 μg/mL (ex-LMSIII). The hydroxyl radical and its subsequent radicals are the most harmful reactive oxygen species, as they are mainly responsible for the oxidative injury in many biomolecules [4,16]. In the method, hydroxyl radicals are produced via the Fenton reaction in the system. The scavenging capacity of hydroxyl radicals in the samples is compared to a positive control consisting of ascorbic acid and Trolox. The scavenging effects of the samples are shown in Table 2. The IC_50_ values of the samples ranged from 49.13 to 69.12 μg/mL. Based on the comparison of the IC_50_ values, the order of the hydroxyl radical-scavenging activity was found to be as follows: ex-LMSIII fraction > ex-LMSII fraction > ex-LMSII fraction (Table 2).

**Table 2:** IC_50_ values in the DPPH, ABTS, and OH radical-scavenging activity assay of ex-LMS I, ex-LMS II, and ex-LMS III isolated from *C*. *unicolor* submerged cultures.

IC_50_ (μg/mL)			
	DPPH radical scavenging	ABTS radical scavenging	OH radical scavenging
ex-LMS I	64.14 ± 2.27a	81.12 ± 3.29a	69.12 ± 1.82a
ex-LMS II	49.22 ± 1.83b	39.78 ± 2.09b	57.94 ± 1.27b
ex-LMS III	20.39 ± 4.17c	31.49 ± 4.91b	49.13 ± 1.34c

All results are expressed as mean ± SD from three experiments (n = 3). Values with different letters within the columns are significantly different (p ≤ 0.05).

The strong antioxidant activity of fungal extracts is most often correlated with high content of total phenols. The present results demonstrate stronger activity of ex-LMSIII than that of the standard antioxidants (ascorbic acid and Trolox). Our results indicate that the analyzed ex-LMS fractions with lower phenol content exert a stronger radical scavenging effect, suggesting that phenols are not the main factor in their antioxidant activity. In our previous study, we demonstrated the antioxidant activity of the ex-LMS starting fraction of *C*. *unicolor*. The scavenging abilities of *C*. *unicolor* ex-LMS at the concentration range of 6.25–800 μg/mL were between 20 and 90% for ABTS and between 10 and 59% for DPPH. The IC_50_ values in the case of the ABTS- and DPPH-scavenging tests were 25.0 μg/mL and 85.3 μg/mL, respectively [33]. To our knowledge, there is no adequate data on *C*. *unicolor* comparable to the data obtained in our work. Literature data confirm the antioxidant activity of non-phenolic substances of fungal origin. Antioxidant metabolites, i.e. 2,4,6-trimethylacetophenone imine, glutamyl tryptophan, azatadine, and lithocholic acid glycine conjugate isolated from *Boletus spp*. exhibited antioxidant activity [50].

### Antibacterial activity of ex-LMSI, ex-LMSII, and ex-LMSIII

A wide range of components derived from mushrooms have been reported to possess antibacterial properties. Our previous studies have shown that total ex-LMS was much more effective towards *E*. *coli* and *S*. *aureus* bacterial cells than the extracellular fraction of laccase or the polysaccharide fraction from *C*. *unicolor* [33]. The activity of ex-LMSI, ex-LMSII, and ex-LMSIII was tested against bacteria, and penicillin sodium was used as a standard drug for comparison. The test microorganisms used in the present studies included *E*. *coli* (Gram-negative bacteria) as well as *S*. *aureus* and *B*. *subtilis* (Gram-positive bacteria). In our experiments, the low-molecular subfractions ex-LMSI, ex-LMSII, and ex-LMSIII showed relatively strong antimicrobial activity against all the Gram-positive and Gram-negative bacterial species used (Tables 3 and 4). The inhibition zone values obtained during the experimental cycle indicate that *S*. *aureus* was the most susceptible bacterium. In turn, *B*. *subtilis* and *E*. *coli* exhibited the lowest sensitivity. The ex-LMSII fraction was the most effective against all the microorganisms tested. These results are in agreement with the reports demonstrating that, in general, Gram-positive bacteria are considered more sensitive to different natural and synthetic compounds than Gram-negative bacteria due to the differences in the structure of their cell walls [4,51]. The cell wall of Gram-positive bacteria is composed of peptidoglucans and teichoic acids, while the cell wall of Gram-negative bacteria is composed of peptidoglucans, lipopolysaccharides, and lipoproteins [52–53]. This observation is in agreement with other studies [20,54] that have demonstrated greater sensitivity of Gram-positive bacteria than that of Gram-negative bacteria. The results described above are similar to those presented in this report.

**Table 3:** Antibacterial activities (inhibitory zone assay) of ex-LMSI, ex-LMSII, and ex-LMSIII (1mg/mL) isolated from *C*. *unicolor* submerged cultures.

	Diameters of the inhibition zones (mm)		
	*E*. *coli*	*S*. *aureus*	*B*. *subtilis*
ex-LMS I	1.68 ± 0.16ab	2.38 ± 0.14a	1.69 ± 0.11a
ex-LMS II	1.96 ± 0.12a	3.21 ± 0.23b	2.76 ± 0.19b
ex-LMS III	1.55 ± 0.14b	2.36 ± 0.30a	2.76 ± 0.19b
physiological saline	-^a^	-^a^	-^a^
penicillin sodium^b^	2.86 ± 0.17c	3.08 ± 0.26b	3.01 ± 0.16b

All results are expressed as mean ± SD from three experiments (n = 3). Values with different letters within the columns are significantly different (p ≤ 0.05).

^a^Not detected.

^b^The concentration of penicillin sodium was 0.5 mg/well for the Gram-negative microorganisms and 0.25 mg/well for the Gram-positive bacteria.

**Table 4:** Antibacterial activities (minimum inhibitory concentration) of ex-LMSI, ex-LMSII, and ex-LMSIII (1mg/mL) isolated from *C*. *unicolor* submerged cultures.

	Minimum inhibitory concentration (mg/mL)		
	*E*. *coli*	*S*. *aureus*	*B*. *subtilis*
ex-LMS I	50.00a	12.50a	25.00a
ex-LMS II	100.00b	6.25b	12.50b
ex-LMS III	100.00b	50.00c	25.00a

All results are expressed as mean ± SD from three experiments (n = 3). Values within the column are significantly different (p ≤ 0.05).

The MIC values for the ex-LMS fractions against *B*. *subtilis*, *S*. *aureus*, and *E*. *coli* showed that ex-LMSI and ex-LMSII exerted a higher inhibition effect on *S*. *aureus* than on *B*. *subtilis* and *E*. *coli* (Tab. 4). The ex-LMSII fraction was the most effective as previously. The MIC values for this fraction against *B*. *subtilis*, *S*. *aureus*, and *E*. *coli* were 12.5, 6.25, and 100 mg/mL, respectively.

These results suggest that the extracellular low molecular weight secondary metabolites derived from the *C*. *unicolor* culture fluid, especially ex-LMSII, may be an interesting source of antibacterial substances.

### Anticancer activity of ex-LMSI, ex-LMSII, and ex-LMSIII

**Fig 2:**
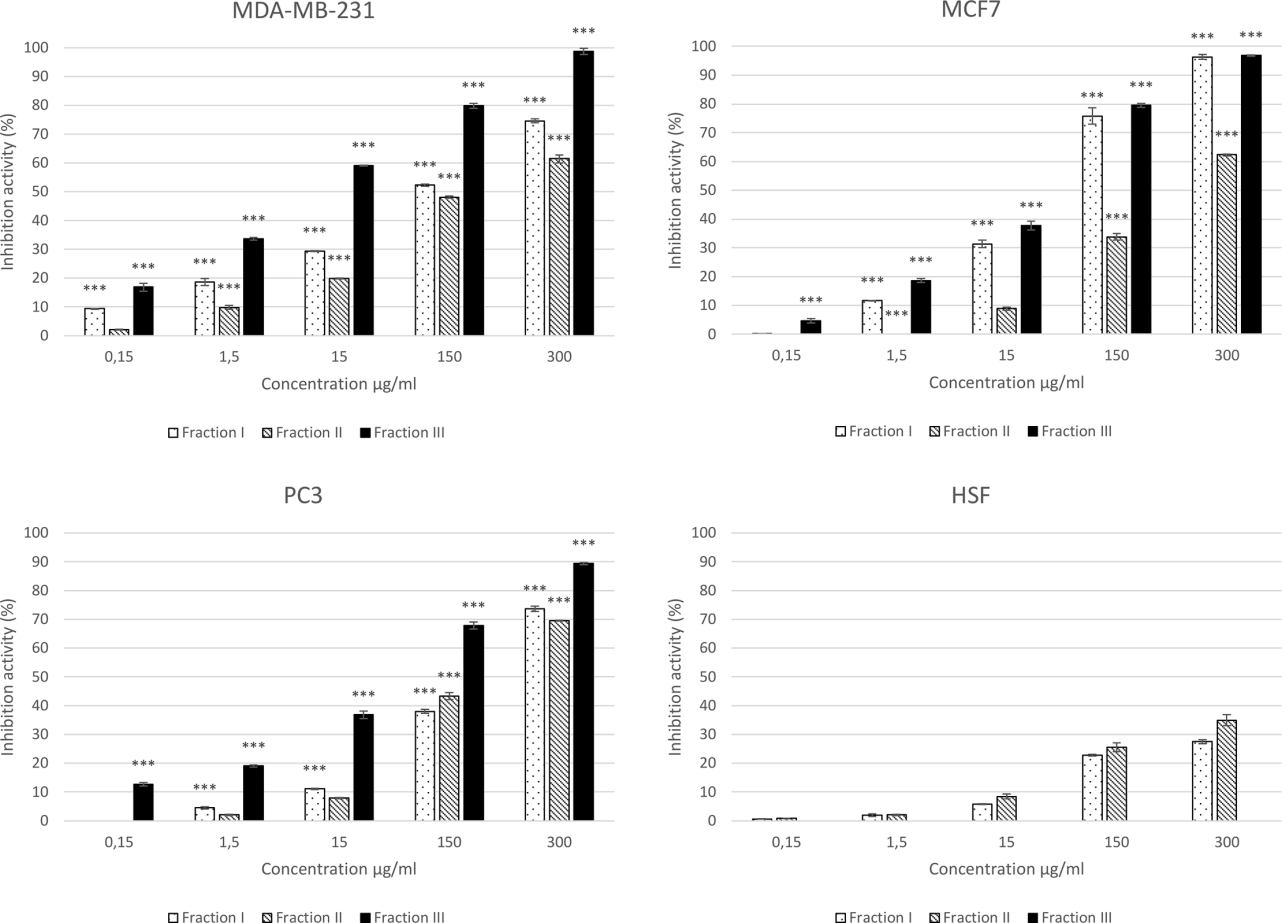
Cytotoxic effect (inhibition activity of ex-LMSI, ex-LMSII, and ex-LMSIII isolated from *C*. *unicolor* towards three cancer cell lines MCF7, MDA-MB-23, and PC3 as well as the HSF line). The MTT assay was performed after 48 h of incubation. Values are mean ± SD (n = 3).

Approximately 651 species of higher basidiomycetes with anticancer activity have been described in the literature [55]. For example, an ethanol extract of *Antrodia cinnamomea* mycelia was found to possess high antihepatoma activity. Isolation of antiproliferative metabolites from fungal cultures and investigation of their mechanism of action may lead to the development of a new class of anticancer drugs [56]. In our previous studies, we have shown that the *C*. *unicolor* ex-LMS total fraction has antiproliferative properties toward murine melanoma B16-F10 cells [31]. To study the growth inhibitory activity of the ex-LMS fractions from *C*. *unicolor in vitro*, human cancer cell lines (MCF7, MDA-MB-231, and PC3) were incubated with various concentrations of ex-LMSI, ex-LMSII, and ex-LMSIII (Fig 2).

The potentially toxic effect of the investigated subfractions was assessed using the MTT assay (Table 5). The inhibition of the viability of all these three human cancer cells by ex-LMSI, ex-LMSII, and ex-LMSIII affected the normal human fibroblast cells in a very negligible way. The highest percentage of inhibition was obtained at a concentration of 300 μg/mL over all the examined tumor cell lines, and ex-LMSIII proved to be the most effective.

**Table 5:** Cytotoxic IC_50_ of ex-LMSI, ex-LMSII, and ex-LMSIII isolated from *C*. *unicolor* against cancer cells.

	IC_50_ (μg/mL)			
	ex-LMSI	ex-LMSII	ex-LMSIII	Paclitaxel (positive control)
PC3	97.17 ± 3.23a	103.32 ± 6.71a	60.66 ± 4.67b	11.73 ± 7.14c
MDA-MB-231	74.91 ± 4.51a	89.85 ± 3.89b	52.25 ± 6.48c	14.38 ± 2.30d
MCF7	66.22 ± 3.85a	109.45 ± 6.49b	54.92 ± 2.27c	nd*
HSF	>100	>100	>100	nd*

All results are expressed as mean ± SD from three experiments (n = 3). Values with different letters within the rows are significantly different (p ≤ 0.05).

* Not detected.

Literature data indicating the antitumor activity of fungi are primarily focused on the use of mycelium extracts. For example, Liu et al. demonstrated the cytotoxic activity of a *Ramaria flava* ethanolic extract against human cancer cell lines (BGC-803, NCI-H520, and MDAMB-231) [4]. They demonstrated that the ethanol extract had the strongest growth inhibitory activity against human breast cancer cells of the MDA-MB-231 line (IC_50_ = 66.54 ± 4.27 μg/ml), and the inhibition rate was 71.66% at a concentration of 200 μg/ml. The *in vitro* anticancer activity of ethanol extracts was also confirmed by Wu et al. investigating the effects of *Fomitopsis pinicola*, *Ganoderma sinense*, *Fomitopsis officinalis*, and *Polyporus melanopus* extracts on cancer cell lines HepG2 and S-180 [57]. In our study, a significant percentage (59.08%) of ex-LMSIII inhibition of the MDA-MB-231 tumor line was observed at a concentration of 15 μg/ml, which did not affect normal human fibroblast cells. As shown by the literature data, most studies related to the antitumor properties of fungi focus on alcoholic or aqueous extracts or substances isolated from the mycelium. In our study, we used a low molecular weight fraction of the post-culture fluid obtained in large quantities during liquid mushroom culture. The fraction has not been used in investigations of fungal antitumor activity to date. The omission of organic extraction in the separation procedure is also very important especially from the biomedical point of view. All these results suggest that the ex-LMSI, ex-LMSII, and ex-LMSIII subfractions can be a rich source of highly bioactive substances, especially with cytotoxic potential.

## Conclusions

It was found in the present study that the three bioactive subfractions of low molecular weight secondary metabolites: ex-LMSI, ex-LMSII, and ex-LMSIII isolated from *C*. *unicolor* culture fluid possessed anticancer, antioxidant, and antibacterial properties. Our results show that post-culture medium containing secondary metabolites of less than 10 kDa can be a rich source of natural anticancer, antioxidant, and antibiotic substances and can be important in the control of various human and animal diseases. These results suggest that *C*. *unicolor* can be an effective source of pharmaceuticals.
